# Supplementary material for: Cord blood IgA/M reveals in utero response to SARS-CoV-2 with fluctuations in relation to circulating variants
Source: Nat Commun. 2025 Apr 14;16:3551. doi: 10.1038/s41467-025-58768-5 (PMC11997084; doi:10.1038/s41467-025-58768-5)
Supplement: Supplementary file 1 — Supplementary Information [file 41467_2025_58768_MOESM1_ESM.pdf]

SUPPLEMENTARY TABLES

SUPPLEMENTAL TABLE LEGENDS

Table S1: Limit of Detection (LoD) and Limit of Blank (LoB) of IgA and IgM detection assay for anti-RBD (Receptor Binding Domain) and anti-N (Nucleocapsid) among 103 cord bloods collected before 2019. Monte Carlo simulation was conducted to estimate the false positive rate. Using a Gamma distribution with shape parameter α and scale parameter θ, 10,000 random samples were generated, each with a sample size of 100,000. The mean false positive rate across all samples was calculated and reported as the overall false positive rate. Additionally, the 2.5th and 97.5th percentiles of the false positive rates were determined to establish the 95% CI. The random number seed was set using call streaminit (12345) in SAS to ensure reproducibility.

Table S2. Median IgG anti-RBD (Receptor Binding Domain) level among samples with detectable SARS-CoV-2 IgM and/or IgA by Isotype Combination: IgM+ only, IgA+ and IgM+, and IgA+ only (N=295)

Wilcoxon rank-sum tests evaluated between group difference. Kruskal-Wallis test evaluated overall differences among the three groups. To account for multiple comparisons, the Bonferroni correction method was used which multiplies the raw P values by the number of tests (n=3). The statistics used were all two-sided.

Table S3. Median IgG anti-N (Nucleocapsid) level among samples with detectable SARS-CoV-2 IgM and/or IgA by Isotype Combination: IgM+ only, IgA+ and IgM+, and IgA+ only (N=295)

Wilcoxon rank-sum tests evaluated between group difference. Kruskal-Wallis test evaluated overall differences among the three groups. To account for multiple comparisons, the Bonferroni correction method was used which multiplies the raw P values by the number of tests (n=3). The statistics used were all two-sided.

Table S1. Limit of Detection (LoD) and Limit of Blank (LoB) of IgA and IgM detection assay for anti-RBD (Receptor Binding Domain) and anti-N (Nucleocapsid) among 103 cord bloods collected before 2019

	Anti-RBD		Anti-N	
	IgA	IgM	IgA	IgM
Median	3.00	6.00	3.00	15.00
[range]	[1-6]	[2-45]	[1-9]	[3-53]
[1st Quartile – 3rd Quartile]	[2-3]	[5-8]	[2-3]	[11-21]
Mean ($\alpha\theta$)	2.89	7.70	2.82	17.32
[Standard Deviation (SD)]	[1.17]	[5.69]	[1.38]	[9.19]
Variance ($\alpha\theta^2$)	1.36	32.34	1.89	84.41
Limit of Blank	4.80	17.06	5.08	32.43
Limit of Detection (LoB + 1.645 x SD)	6.72	26.41	7.34	47.55
False Positive Rate	0.52%	1.03%	0.64%	0.71%
95% CI	(0.48%, 0.57%)	(0.97% ,1.09%)	(0.61%,0.71%)	(0.67%, 0.77%)
Limit of Detection (LoB + 5xSD)	10.63	45.5	11.96	78.37
False Positive Rate	0.001%	0.016%	0.003%	0.004%
95% CI	(0%, 0.004%)	(0.009%, 0.025%)	(0%, 0.006%)	(0.001%, 0.008%)

The parameters of a Gamma distribution α and θ were calculated from Mean ($\alpha\theta$) and Variance ($\alpha\theta^2$) of the sample

Table S2. Median IgG anti-RBD (Receptor Binding Domain) among samples with detectable SARS-CoV-2 IgM and/or IgA by Isotype Combination: IgM+ only, IgA+ and IgM+, and IgA+ only (N=295)

Isotype	n	IgG anti-RBD MFI (Median [IQR])	Comparison	p-value
IgM+	31	8140 [11512]	IgA+	0.0003
IgA+	224	15733 [8448]	IgM+ IgA+	0.0363
IgM+ IgA+	40	12620.50 [9610]	IgM+	0.1959
			Overall p-value	<0.0001*

MFI= Median Fluorescence Intensity, IQR=Inter Quartile Range. IgM positive only denoted by 'IgM+'; both IgA and IgM positive denoted by 'IgA+ IgM+'; IgA positive only denoted by 'IgA+'

Table S3. Median IgG anti-N (Nucleocapsid) among samples with detectable SARS-CoV-2 IgM and/or IgA by Isotype Combination: IgM+ only, IgA+ and IgM+, and IgA+ only (N=295)

Isotype	n	IgG anti-N MFI (Median [IQR])	Comparison	p-value
IgM+	31	1347 [2124]	IgA+	0.0018
IgA+	224	2809.5 [7496]	IgM+ IgA+	0.1776
IgM+ IgA+	40	1779 [5133]	IgM+	0.4224
			Overall p-value	0.0008

MFI= Median Fluorescence Intensity, IQR=Inter Quartile Range; IgM positive only denoted by 'IgM+'; both IgA and IgM positive denoted by 'IgA+ IgM+'; IgA positive only denoted by 'IgA+'

SUPPLEMENTARY FIGURES

SUPPLEMENTAL FIGURE LEGENDS

Figure S1: Evaluation of SARS-CoV-2 IgM and IgA in pre-2019 Cord Blood Samples (CBS)

A and B: Evaluation of pre-SARS-CoV-2 Cord Blood Samples to Determine Threshold for IgM and IgA. Using 103 remnant CBS collected before 2019, expected to be true negatives, we calculated anti-RBD (Receptor Binding Domain) and anti-N (Nucleocapsid) background signals for IgM (**panel A**, in green) and IgA (**panel B,** in pink) isotypes to establish threshold (solid lines) as described in the Methods section. We set the Limit of Detection (LoD) at Limit of Blank LOB): $LoB+5 \times SD_{blank}$ rather than the standard $LoB+1.645 \times SD_{blank}$ to ensure a conservative estimation and optimize test specificity. The final thresholds are shown as Median Fluorescent Intensity (MFI).

Figure S2: Evaluation of SARS-CoV-2 IgM and IgA in Cord Blood Samples (CBS)

A: IgM anti-N (Nucleocapsid) **and anti-RBD** (Receptor Binding Domain) were measured in 1035 samples. Positivity thresholds established in panel A for each target are indicated in green lines. **B: IgA anti-N and anti-RBD** were measured in 1038 samples. Positivity thresholds established in panel B for each target are indicated in pink lines.

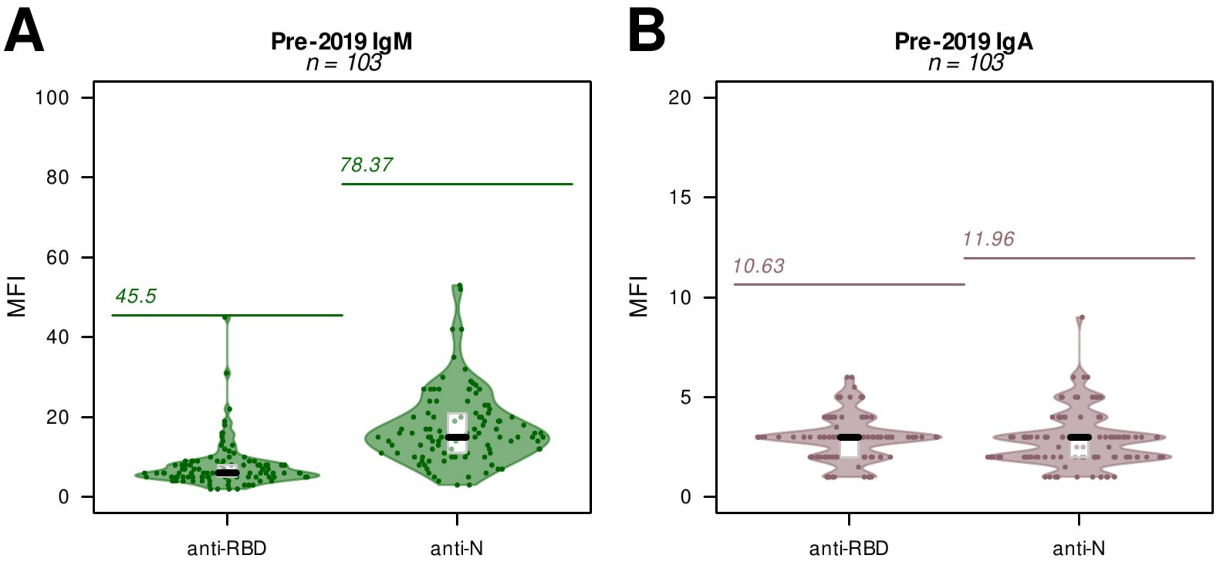

Figure S1: Evaluation of IgM and IgA in Pre-2019 Cord Blood Samples

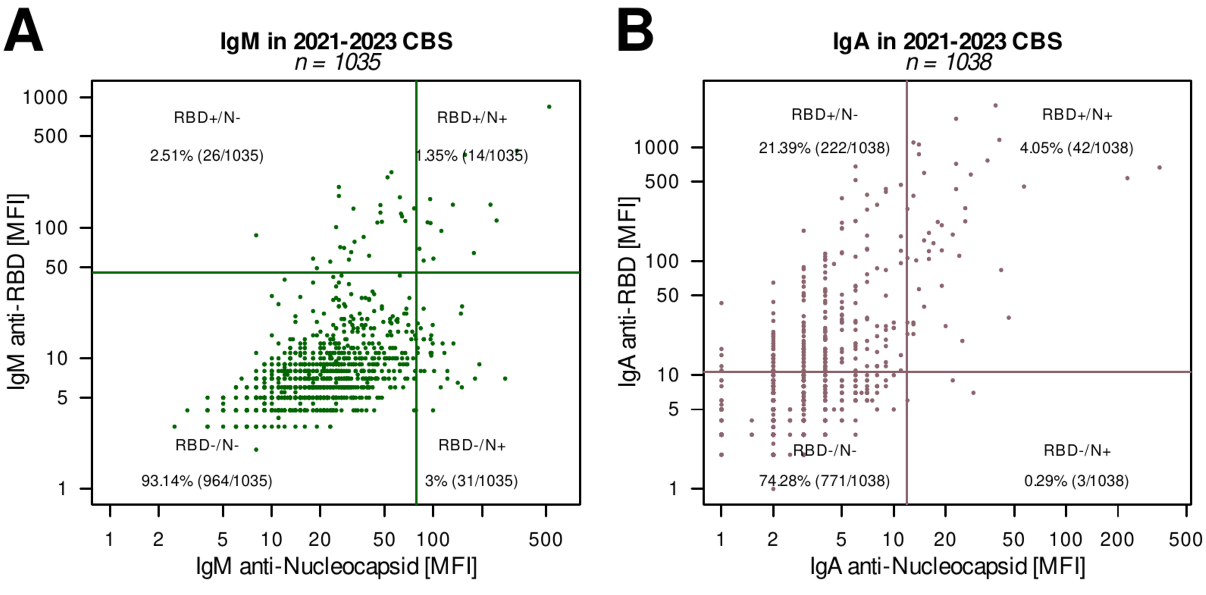

Figure S2: Evaluation of IgM and IgA in Cord Blood Samples
